# Influence of Parameters on Photodynamic Therapy of Au@TiO_2_–HMME Core-Shell Nanostructures

**DOI:** 10.3390/nano12081358

**Published:** 2022-04-15

**Authors:** Ping Wang, Luwei Zhang, Zhenxi Zhang, Sijia Wang, Cuiping Yao

**Affiliations:** Key Laboratory of Biomedical Information Engineering of Ministry of Education, Institute of Biomedical Photonics and Sensing, School of Life Science and Technology, Xi’an Jiaotong University, Xi’an 710049, China; wangping007@stu.xjtu.edu.cn (P.W.); lwzhang@xjtu.edu.cn (L.Z.); zxzhang@mail.xjtu.edu.cn (Z.Z.); wang_sijia@xjtu.edu.cn (S.W.)

**Keywords:** Au nanoparticles, TiO_2_, HMME, conjugate, PDT

## Abstract

Photodynamic therapy (PDT) is a promising tumor therapy and has been proven to be an effective, safe and minimally invasive technique. Hematoporphyrin monomethyl ether (HMME) mediated PDT has been used in clinical treatment of port wine stain (PWS) due to its single component, high yield of singlet oxygen and short light-sensitive period. However, as an amphiphilic photosensitizer, HMME is easy to aggregate due to the presence of a hydrophobic group, which undesirably reduced its generation of singlet oxygen and bioavailability. In this study, we synthesized the stable conjugate of Au@TiO_2_ core-shell nanostructure with HMME, and the influence of different factors on PTD efficiency were studied. The results showed that the nanostructure had higher PTD efficiency for KB cells than that of HMME. The irradiation wavelength, gold nanoparticle shape and the shell thickness are all important factors for KB cell PDT.

## 1. Introduction

Continuous optimization and development in surgery, radiotherapy [1,2], chemotherapy [3], gene therapy [4,5] and photodynamic therapy (PDT) [6,7,8,9,10] demonstrate that scientists are continuing the fight against the most serious threat to human health—malignant tumors. At present, all these available treatment strategies used in clinical treatments inevitably cause damage to normal tissues and severe undesired side effects while killing tumor cells and/or tissues. Therefore, it is urgent to find a treatment strategy that not only selectively kills cancer tissues but also relatively minimizes the damage to surrounding normal tissues. For this purpose, the advantages of photodynamic therapy are more significant [11], especially for superficial cancers, such as skin cancer and oral mucosa cancer.

Photosensitizer [12,13], excitation light and reactive oxygen species (ROS) are the three main elements in photodynamic therapy [14]. The light of a specific wavelength interacts with the photosensitizer, which induces a series of cellular and tissue effects. Photosensitizers exposed directly to light will realize the transfer of photon energy to molecular oxygen, producing ROS and singlet oxygen to induce apoptosis and necrosis in the process [14]. Obviously, a photosensitizer with stable performance, strong photosensitivity, low toxicity and fast metabolism is the key to improve the PDT effect. Hematoporphyrin monomethyl ether (HMME) is such a photosensitizer with the promising clinical application prospect [15,16,17]. HMME mediated PDT has been used in clinical treatment of port wine stain (PWS) [18] and glioma due to its single component, high yield of singlet oxygen and short light avoidance period after drug delivery, which has an evidently significant effect [16]. However, as an amphiphilic photosensitizer, HMME is comprised of easy to form aggregates due to the presence of hydrophobic groups, which reduces its photosensitive viability or PDT efficiency.

Using designed nanodrug delivery systems to improve the delivery efficiency and singlet oxygen generation of a photosensitizer is one of the promising strategies [19,20]. Recently, nanomaterials such as liposomes [21,22], natural polymer nanoparticles [23,24], metal-based nanoparticles [25,26,27,28], quantum dots [29], etc., have been widely used to improve the effect of PDT. Among these, gold nanoparticles (GNPs) [30,31,32] stand out because of their unique optical characteristics, easily modified chemical properties and good biocompatibility. Additionally, GNPs can enhance the ROS and singlet oxygen generation of photo-agents adsorbed on their surface [33]. However, the direct exposure of photo-agents to the physiological environment usually leads to dissatisfactory drug loading or unexpected drug inactivation [34], and when using GNPs as drug carriers, the spatial distance between the photo-agents and the surface of nanoparticles will influence the optical properties of the agents due to the localized surface plasma resonance (LSPR) of GNPs. When the photo-agents are tightly combined on the surface of GNPs, their fluorescence will be quenched and the generation of ROS and singlet oxygen will reduce because of the resonance energy transfer. Conversely, when the spatial distance exceeds a certain range, the LSPR effect of GNPs will be too weak to enhance the singlet oxygen of photo-agents.

Nanosized titanium dioxide (TiO_2_, mainly anatase) is a typical biocompatible semiconductor oxide material, which has been widely used in photocatalysis [35], dye-sensitized solar cells (DSSCs) [36], nano-therapeutic drugs [37,38], photocatalyse water splitting [39], organic degradation [40] and other aspects [41]. In this study, we designed to cover the GNP’s surface with a certain thickness of TiO_2_ layer to form an Au@TiO_2_ core-shell structure, which could not only improve the photosensitizer loading by utilizing the abundant hydroxyl groups in TiO_2_ shell but also maximize the enhancement of LSPR to improve the intracellular uptake of the photosensitizer and PDT efficiency. A complete experimental system using the conjugate (AuNP@TiO_2_–HMME and AuNR@TiO_2_–HMME) was established, and the photodynamic effect of the conjugate on KB cells was tested in this study, with a view that the verified results were expected to provide references for improving the development of drug delivery system for PDT.

## 2. Material and Methods

### 2.1. Preparation and Modification of Gold Nanoparticles

The gold nanoparticles obtained by the seed growth method [42,43] were coated with a layer of surfactant cetyl trimethyl ammonium bromide (CTAB). The CTAB bound to the surface of the gold nanoparticles would form a chemically adsorbed bilayer, hindering the binding to other biomolecules. Therefore, it is indispensable to make the gold nanoparticles have better biocompatibility, stability and lower cytotoxicity by modifying the surface. In this process, Poly-(sodium 4-styrenesulfonate) (PSS) was obtained from Sigma Aldrich Co. (St. Louis, MO, USA) and employed to modify the gold nanospheres owing to its low toxicity and strong anion electrolytic activation [44]. The specific process was to take 10 mL of the prepared gold nanospheres solution at 27 °C, centrifuge at 13,000 rpm for 20 min, remove excess surfactant CTAB and resuspend in a certain volume of ultrapure water to make the absorbance OD ≈ 1. Then, 0.8775 g NaCl was dissolved in 50 mL ultrapure water to obtain 50 mL, with 0.3 mol/L NaCl solution as the original solution, taking a part to dilute to 6 mmol/L. 0.02 g PSS which was dissolved in 10 mL, 6 mmol/L NaCl solution, and magnetic stirring was performed to obtain 10 mL, 2 g/L PSS solution. During magnetic stirring, 10 mL of gold nanospheres solution (the absorbance OD ≈ 1) was added dropwise to the PSS solution, and the stirring time was at least 4 h, so that the PSS could be fully absorbed on the surface of gold nanospheres. After completion, the solution was left standing for 4 h at 27 °C. The prepared PSS–AuNP solution was centrifuged at 10,000 rpm for 20 min, repeated to remove excess PSS from the solution, and then the solution was dissolved in 0.2 mL of ultrapure water for use.

Rod-shaped gold nanoparticles (gold nanorods) as the anisotropic precious metal nanoparticles specifically have two plasmon resonance absorption peaks, which are the transverse absorption peak (SPR_T_) and the longitudinal absorption peak (SPR_L_). The position of the SPR_L_ absorption peak is highly adjustable, which can be adjusted from the visible light region to the near infrared region. The special properties bring a lot of biological applications [45], which are very notable. Based on the scheme of preparing gold nanorods by Hongwei et al. [46], gold nanorods were prepared and were further oxidized to shrink the rods to produce gold nanorods with the desired SPR_L_ [47]. In this study, based on successful preparation of gold nanorods (in practice, SPR_L_ = 810 nm, aspect ratio = 3.86), heating at 60 °C water bath, 25 mL,1 mol/L HCl and 0.25 mL, 0.5% H_2_O_2_ were added to the solution under magnetic agitation in order to obtain gold nanorods with blue-shifted absorption peaks (in practice, SPR_L_ = 600 nm, aspect ratio = 1.97).

### 2.2. Synthesis of the Au@TiO_2_–HMME Conjugates

**Figure 1:**
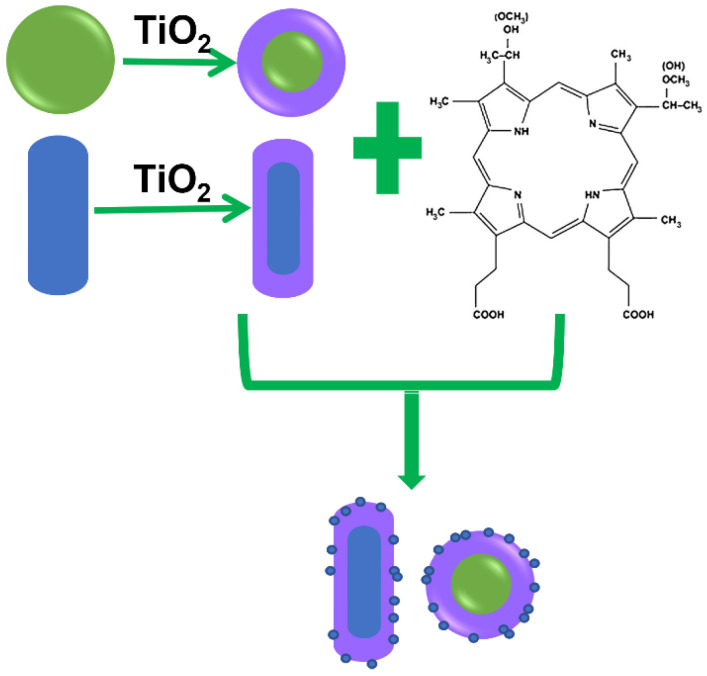
Flowchart of preparation of Au@TiO_2_–HMME.

The process of the Au@TiO_2_–HMME conjugate preparation is shown schematically in Figure 1. The TiOH^2+^ generated by the hydrolysis of TiCl_3_ could closely attach to negatively charged PSS through electrostatic attraction and further be oxidized to obtain AuNP@TiO_2_ core-shell nanostructures; after that, the AuNP@TiO_2_ core-shell nanostructures were mixed with HMME to form a conjugate in which the TiO_2_ shell thickness is determined by the NaHCO_3_ amount. Briefly, 1.5 mL, 0.93 mol/L of NaHCO_3_ solution was dropwise added to the Erlenmeyer flask containing 8 mL of ultrapure water and 0.3 mL of TiCl_3_ solution with stirring. When the solution became darker, the prepared PSS–AuNP solution was added immediately and the reaction maintained for 30 min. The obtained AuNP@TiO_2_ solution was centrifuged at 8000 rpm for 30 min and repeated to remove excess TiO_2_. Then, the prepared AuNP@TiO_2_ solution (1 nmol/L) was mixed with HMME of different concentrations for reaction at room temperature in the dark for 24 h. The preparation of AuNR@TiO_2_–HMME conjugate was similar to the AuNP@TiO_2_–HMME process.

### 2.3. Characterization of the Au@TiO_2_ Core-Shell Nanostructures and Conjugates

The spectra of HMME, Au nanoparticles, Au@TiO_2_ core-shell nanostructures and Au@TiO_2_–HMME were measured and compared by ultraviolet-visible spectrophotometer (V-550 UV/VIS, JASCO, Ashikaga, Japan).

The morphological characteristics and dispersion of different nanostructures, such as, gold nanospheres, AuNP@TiO_2_ with different size, AuNR@TiO_2_ with different SPR_L_ and the Au@TiO_2_–HMME conjugate, were observed by transmission electron microscopy (TEM) (H-600 TEM, NEC, Tokyo, Japan) The nanomeasure software was used to measure the statistical size of gold nanoparticles. The particle size and surface potential of nanomaterials were measured and analyzed by a laser particle size analyzer (Mastersizer 2000, Malvern, Malvern, UK).

### 2.4. Cell Culture

In this study, human oral epidermoid carcinoma cells (KB cells) purchased from Sigma Co. (Xi’an, China) were used. The culture medium was prepared by adding 89% high glycemic solution DMEM (Hyclone Co., Logan, UT, USA) to 10% fetal bovine serum solution (Hangzhou Sijiqing Bioengineering Materials Co. LTD, Hangzhou, China) and 1% double antibody solution (Haimen Biyuntian Biotechnology Research Institute, Haimen, China). KB cells were incubated with 10 mL culture medium in a cell incubator (Heraell150, Thermo, Waltham, MA, USA), in which the temperature was maintained at 37 °C, the CO_2_ concentration was 5%, and the air humidity was 95%. All the cells used in the experiment were in logarithmic growth phase.

### 2.5. Evaluating the Intracellular Uptake of the Au@TiO_2_–HMME Conjugates

Under the excitation light of 380 nm band, HMME can emit fluorescence with the wavelength of 631 nm and 692 nm. To visually verify the delivery efficiency of the conjugate, after cell lysis with cell lysis buffer (SDS, Haimen Biyuntian Biotechnology Research Institute, Haimen, China), the fluorescence intensity of HMME at 631 nm was measured by a fluorescence spectrophotometer (F-4500, Hitachi, Tokyo, Japan) to determine the amount of HMME entering the cell. Samples were divided into four groups: DMEM solution only, the core-shell nanostructure group (10 nm TiO_2_), HMME only and the conjugate of different TiO_2_ layer thickness (4 nm, 10 nm and 18 nm). The gold nanospheres concentration in each drug group was 0.2 nmol/L, and the HMME concentration was 3 µg/mL.

### 2.6. Evaluating the Photodynamic Effects of the Au@TiO_2_–HMME Conjugates on KB Cells under Different Light Sources

KB cells were used as the research object and Cell Counting Kit-8 (CCK-8) cytotoxicity detection reagent was used to detect the cell viability with HMME and different Au@TiO_2_–HMME conjugates on KB cells. All cell viability results were measured by the absorption at 450 nm 24 h after being mixed with 20 µL CCK-8 reagent using the microplate reader (Infinite 200PRO, Tecan, Männedorf, Switzerland).

In order to determine the reasonable concentration range of HMME in subsequent photodynamic experiments, we first investigated the dark toxicity of different concentration of HMME on KB cells. Then, in order to determine the applicable range for the concentration of gold nanospheres, we studied the effect of AuNP@TiO_2_ core-shell nanostructures with different TiO_2_ shell thickness on cell viability at different gold nanospheres concentration under dark.

The effect of different light source, different TiO_2_ layer thickness and different conjugates on KB cell viability were compared in further detailed experiments. Different illumination light sources were selected, namely a 532 nm LED green light coupled to the optimal excitation wavelength (550 nm) of the conjugate AuNP@TiO_2_–HMME, a xenon lamp with wide spectral range and a 635-nm continuous laser coupled to a characteristic peak (660 nm) of the conjugate AuNR@TiO_2_–HMME.

To compare the effect of light dose, the irradiation dose of a LED green light and a xenon lamp were set as 0.3 J/cm^2^, 0.6 J/cm^2^ and 1.2 J/cm^2^, respectively. The influence of different core-shell thickness of AuNP@TiO_2_–HMME on the PDT effect was studied under the appropriate illumination condition. Finally, the PDT effects of the two conjugates AuNP@TiO_2_–HMME and AuNR@TiO_2_–HMME were compared.

### 2.7. Statistical Analysis

All results of statistical analysis were performed using SPSS 12.0 (IBM, New York, NY, USA). Error bars represent Standard Error of Mean (SEM). *p*-values compared with control group were calculated using paired Student’s *t* test. *, *p* < 0.05. **, *p* < 0.01. ***, *p* < 0.001. ****, *p* < 0.0001. *p*-values of < 0.01 were considered significant, and *p*-values of <0.001 were considered highly significant.

## 3. Results and Discussion

### 3.1. TEM Characterization of the Samples

**Figure 2:**
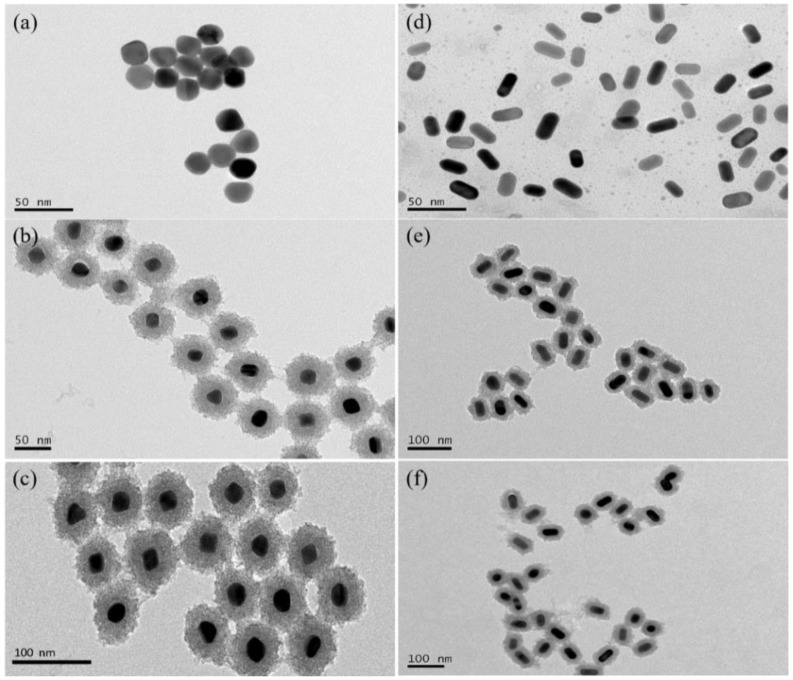
TEM characterization of (**a**) gold nanospheres with the absorption peak of 540 nm (**d**) gold nanorods with the SPR_L_ = 600 nm; The TEM images of different core-shell nanostructures form ((**b**) and (**e**)), and conjugates ((**c**) and (**f**)) were synthesized by different core structures.

Figure 2a–c respectively showed the TEM images of the prepared gold nanospheres with the absorption peak of 540 nm, AuNP@TiO_2_ core-shell nanostructures and the conjugate AuNP@TiO_2_–HMME. The results of particle size statistics by Nanomeasure software also showed that more than 81% of the gold nanospheres are between 21 and 26 nm, which indicated that the gold nanospheres prepared by the seed growth method were uniform in size and stable in shape. The nanostructure with the shell thickness of about 18.23 nm in (b) showed that TiO_2_ was evenly coated on the outer layer of gold nanospheres. After coupling with HMME, the shell thickness of the conjugate AuNP@TiO_2_–HMME is about 18.67 nm in (c). The TEM image of the gold nanorods (SPR_L_ = 600 nm, the aspect ratio = 1.97) that were prepared by the seed growth method and then obtained by oxidizing and shrinking were shown in Figure 2d. Figure 2e is the TEM image of the AuNR@TiO_2_ core-shell nanostructure, in which the longitudinal shell thickness of TiO_2_ was 12.55 nm and the lateral shell thickness was about 13.40 nm. Figure 2f is the TEM image of the conjugate AuNR@TiO_2_–HMME, in which the longitudinal shell thickness of TiO_2_ was about 16.51 nm and the lateral shell thickness was about 17.16 nm. Comparing the TEM images of the conjugate Au@TiO_2_–HMME and the original Au@TiO_2_ core-shell nanostructures, it was found that there were little differences in morphology and size. Subsequently, the spectral absorption peaks of different materials were characterized.

### 3.2. Absorption Spectroscopy Characterization of the Au@TiO_2_ Core-Shell Nanostructures and the Conjugates

**Figure 3:**
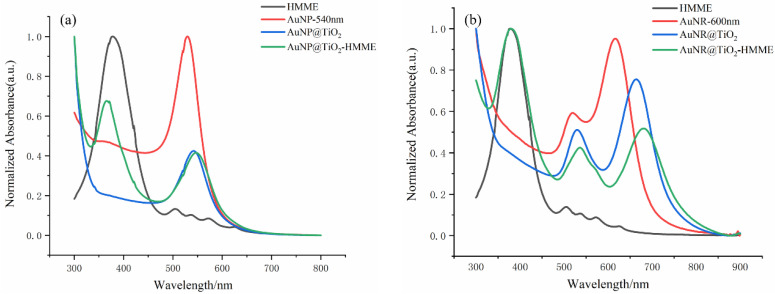
UV-vis spectra (**a**) of HMME, Au nanospheres, AuNP@TiO_2_ core-shell nanostructures and AuNP@TiO_2_–HMME conjugate. UV-vis spectra (**b**) of HMME, Au nanorods, AuNR@TiO_2_ core-shell nanostructures and AuNR@TiO_2_–HMME conjugate.

Figure 3 shows the UV-vis absorption spectra of different materials. Figure 3a shows that coating gold nanosphere with TiO_2_ layers did not seem to affect the absorption peak. Compared with the AuNP@TiO_2_ core-shell structure, the absorption peak of the conjugate was about 5 nm redshift due to HMME binding, indicating that the absorption spectra of the conjugates were not simply superimposed by the spectra of two conjugated components. Gold nanorods with SPR_L_ = 600 nm were used as gold cores, and a layer of TiO_2_ was uniformly coated on the surface to obtain the AuNR@TiO_2_ core-shell nanostructure, the longitudinal absorption peak and the transverse absorption peak of which were redshifted to 664 nm and 530 nm, respectively. After adding equal volume of HMME for 24 h, the longitudinal absorption peak of the AuNR@TiO_2_–HMME complex was redshifted to 676 nm. The results show that the absorption peaks of the conjugates were stable, which was beneficial to the selection of the wavelength of the light source in the subsequent experiments.

### 3.3. Intracellular Uptake of the Au@TiO_2_–HMME Conjugates

**Figure 4:**
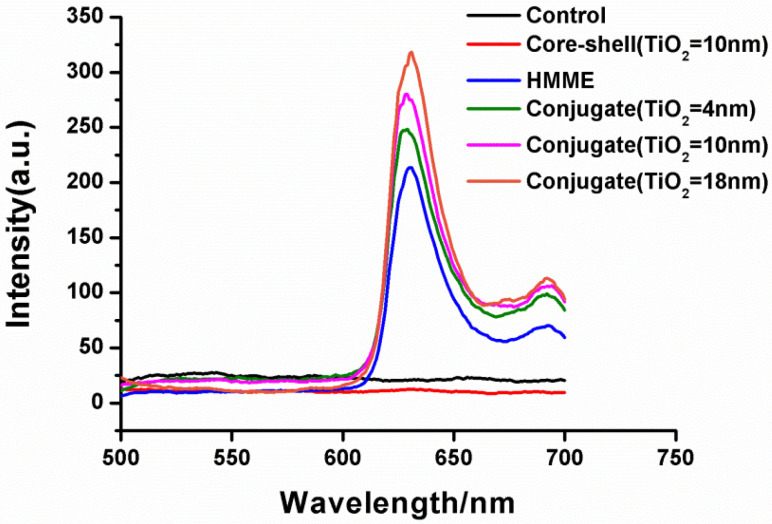
Comparison of intracellular fluorescence intensity of different drug groups after incubation (excitation: 380 nm, emission: 631 nm).

As for the good photodynamic effect of the conjugate on the cells, as mentioned earlier, we speculated that after the amorphous TiO_2_ on the outer layer of the gold nanospheres was combined with HMME, its high specific surface area absorbed more HMME molecules into the cells, which improved the killing effect on the cells. Here, we evaluated the amount of the HMME that the cells absorbed by measurement fluorescence of HMME. Under 380 nm excitation light, that HMME strongly absorbs, the stronger fluorescence (631 nm) intensity was detected. As shown in Figure 4, no fluorescence was produced in the control group and the core-shell nanostructure group. The fluorescence intensity of cells incubated by HMME was lower than that of the conjugate group with the same concentration. Additionally, the fluorescence intensity increased with the increase in TiO_2_ layer thickness of the conjugate, which indicated that with the increase in the thickness of TiO_2_ on the outer layer of the gold nanosphere, the specific surface area of the whole core-shell nanostructure increased so that more HMME molecules could be absorbed into the cell, which is consistent with the previous speculation: namely, the core-shell nanostructure can deliver more photosensitizer molecules into the cell, thus having a more significant killing effect on the cell. Conjugates with different TiO_2_ layer thickness have different cell killing efficiency, which was attributed to the matching degree of the AuNP@TiO_2_ core-shell absorption peak and HMME characteristic peak. The above results can be used as a reasonable temporary explanation for the experiment, and the further mechanism of conjugate binding photosensitizer to promote the efficacy of PDT remains to be explored.

### 3.4. Dark Cytotoxicity of the Au@TiO_2_–HMME Conjugate on KB Cells

**Figure 5:**
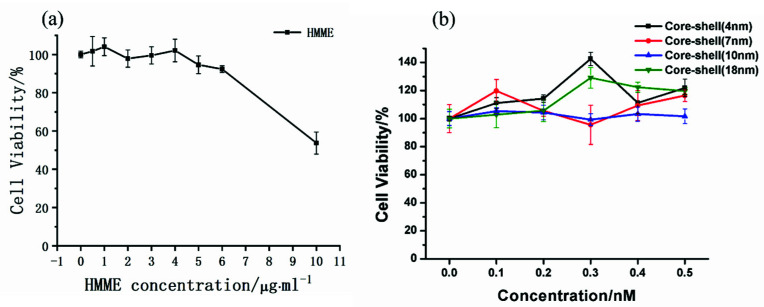
(**a**) Dark toxicity of cells at different concentrations of HMME. (**b**) Dark toxicity of different TiO_2_ shell thickness at different gold nanospheres concentration.

Taking gold nanospheres as an example, the dark toxicity of cells was investigated prior. The dark toxicity of different concentrations of HMME on KB cells are shown in Figure 5a. When the HMME concentration was in 0.5–6 µg/mL, the cell dark toxicity could be ignored with greater than 95% of viability, and when the HMME concentration increased to 10 µg/mL, the cell viability decreased to 53.72% of the control group (DMEM only). In the successive study, the concentration of HMME was reasonably set within 6 µg/mL. Figure 5b shows the dark toxicity results of AuNP@TiO_2_ core-shell nanostructures with different thickness at the concentration of the gold nanospheres solution from 0.1 to 0.5 nmol/L. Compared with the control group (DMEM only), the cell viability was all above 95.55%, indicating that the nanostructure had low dark toxicity to cells. Especially, when the concentration was 0.2 nmol/L, which was chosen as the standard concentration of the gold nanospheres solution for subsequent experiments, the difference was relatively minimal.

### 3.5. Effects of Different Light Sources on PDT of Drug-Treated Cells

**Figure 6:**
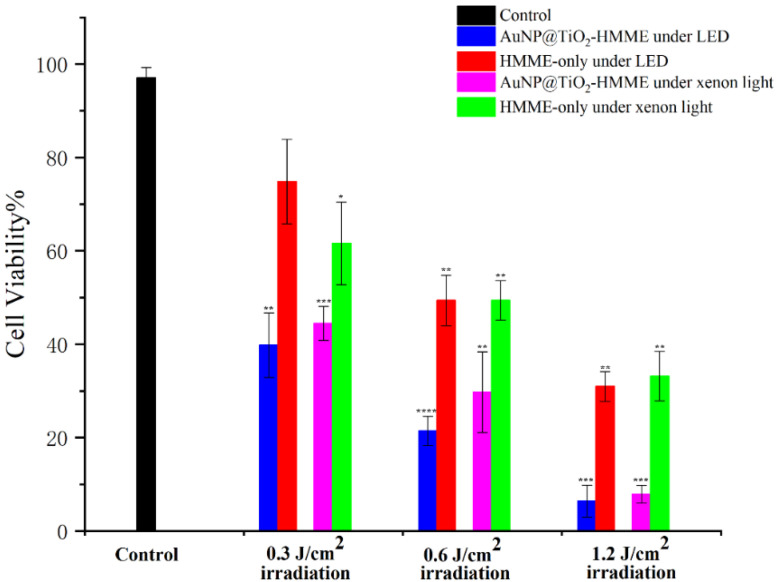
Effect of different light sources and different doses on photodynamic effects of AuNP@TiO_2_–HMME on cells. * was compared with the control group. *, *p* < 0.05. **, *p* < 0.01. ***, *p* < 0.001 and ****, *p* < 0.0001.

The cells incubated with the drug AuNP@TiO_2_–HMME (the shell thickness of AuNP@TiO_2_ was 4 nm and the concentration of HMME is 3 µg/mL) were irradiated by two different light sources (one was green light LED with a central wavelength of 532 nm, and the other was xenon light with a broad spectrum), and the PDT effect is shown in Figure 6, while DMEM-cultured cells were used as the control group. HMME has a characteristic absorption peak at 536 nm, and the characteristic peak of the conjugates is at 540 nm after red shift, which is consistent with the LED wavelength and within the wide spectrum range of xenon lamp. The synergies of PDT of HMME and the surface plasmonic resonance effect should achieve using the wavelength irradiation. When the LED light dose was 0.3 J/cm^2^, the activity of cells incubated by AuNP@TiO_2_–HMME decreased to 39.81% compared with the control group, and the cell killing rate increased by about 35% compared with the 74.85% cell activity of the intergroup control HMME-incubated cell group. Under the same dose of xenon lamp irradiation, the activity of the AuNP@TiO_2_–HMME incubated cell group decreased to 44.45%, and that of the HMME-incubated cell group decreased to 61.57%. Similarly, compared with the control group, the activity of AuNP@TiO_2_–HMME incubated cells decreased to 21.42% and 6.4% under 0.6 J/cm^2^ and 1.2 J /cm^2^ LED irradiation, respectively, while, under the same light dose of xenon lamp irradiation, the activity of the cells incubated by AuNP@TiO_2_–HMME decreased to 29.72% and 7.87%, respectively. Under the conditions of different light doses, 532 nm green LED combined with AuNP@TiO_2_–HMME consistently produced a better photodynamic effect than the xenon lamp. It is also worth noting that under different light sources and different light doses, the conjugated AuNP@TiO_2_–HMME produced significantly higher cell-killing effects than the cell group incubated only with HMME.

### 3.6. Effect of Different Shell Thickness of Conjugates on Cell Photodynamics

**Figure 7:**
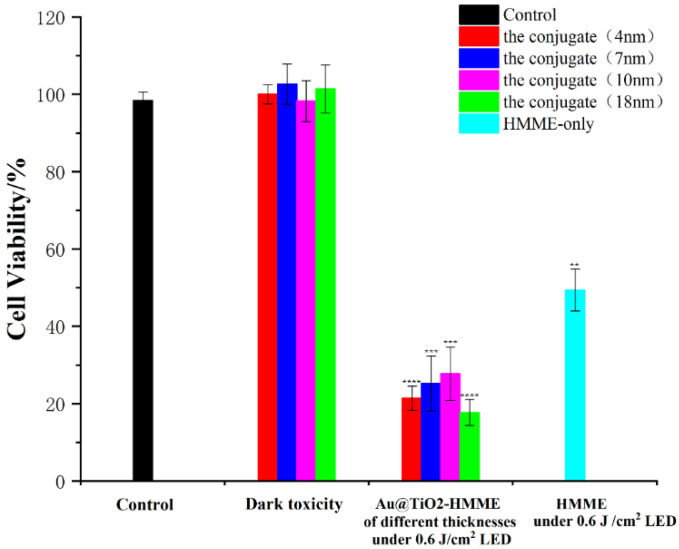
Effect of AuNP@TiO_2_–HMME with different layer thickness on photodynamic action of cells. **, *p* < 0.01. ***, *p* < 0.001 and ****, *p* < 0.0001.

The PH value of solution in the reaction system was increased by adjusting the amount of NaHCO_3_, so as to regulate the thickness of TiO_2_ outer layer of gold nanospheres to 4 nm, 7 nm, 10 nm and 18 nm, respectively. Cells incubated with different drugs were irradiated with 532 nm LED of 0.6 J/cm^2^. Figure 7 showed the photodynamic effect of AuNP@TiO_2_–HMME synthesized by nanocore-shell structures with different shell thickness. Compared with the control group, the activity of cells incubated with AuNP@TiO_2_–HMME conjugates of 4 nm core-shell thickness and 18 nm core-shell thickness decreased to 21.42% and 17.73%. The activity of cells incubated with conjugates of 7 nm core-shell thickness and 10 nm core-shell thickness decreased to 25.24% and 27.77%. By contrast, the conjugates AuNP@TiO_2_–HMME with 4 nm and 18 nm shell thickness seemed to show better photodynamic effects, the reasons for which are discussed later. Under the same conditions, the cell activity of HMME-treated cells decreased to 49.38%, which was much higher than that of the conjugate incubated cells.

### 3.7. Comparison of Photodynamic Effects of Different Core-Shell Nanostructures Combined with HMME

**Figure 8:**
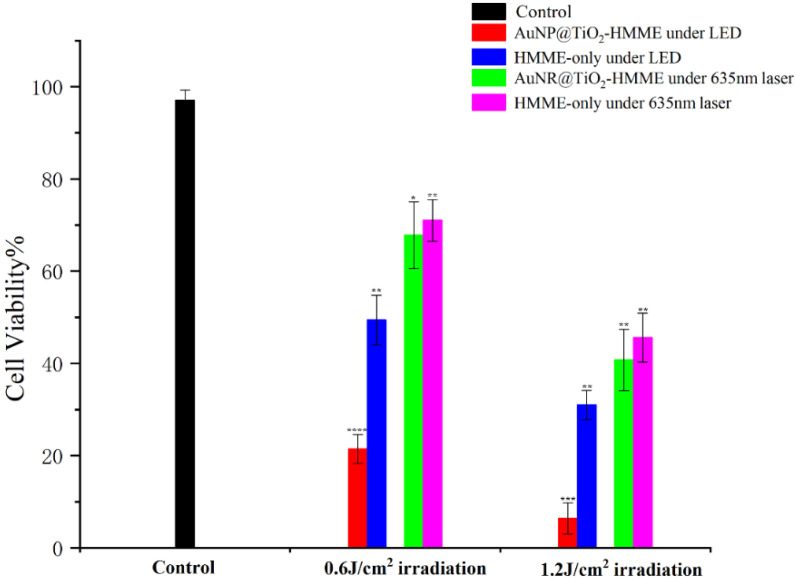
Comparison of photodynamic effects of AuNP@TiO_2_–HMME and AuNR@TiO_2_–HMME on cells. * was compared with the control group. *, *p* < 0.05. **, *p* < 0.01. ***, *p* < 0.001 and ****, *p* < 0.0001.

Figure 8 shows the changes in cell activity after incubating cells with the synthesized conjugates AuNP@TiO_2_–HMME and AuNR@TiO_2_–HMME under different light doses. Using a LED with a central wavelength of 532 nm and a 635-nm laser as the light sources, the cells were then incubated with the synthesis of AuNP@TiO_2_–HMME with an excitation wavelength of about 532 nm and AuNR@TiO_2_–HMME with a SPR_L_ of 630 nm, and the cell survival rate calculated under different light doses. Compared with cells incubated only with HMME, both cell groups incubated with AuNP@TiO_2_–HMME and AuNR@TiO_2_–HMME showed better photodynamic effects. Compared with the control group, the activity of the cell group incubated by AuNP@TiO_2_ decreased to 21.42% at 0.6 J/cm^2^ irradiation, while the cell activity of the AuNR@TiO_2_–HMME incubated group only decreased to 67.79%. When the light dose was increased to 1.2 J/cm^2^, the cell activity decreased to 6.4% and 40.74%, respectively. The experimental results showed that the core-shell nanostructures synthesized by using gold nanorods as the core and being coated by TiO_2_ and the core-shell nanostructures synthesized by using gold nanospheres as the core were combined with HMME, in which the latter obviously showed better photodynamic effect.

## 4. Discussion

In this study, we prepared a series of Au@TiO_2_ core-shell nanostructures to evaluate their enhancement for HMME mediated PDT on KB cells. Evaluation of the PDT effect of the Au@TiO_2_–HMME conjugates was shown in Figure 6, Figure 7 and Figure 8. The first certainty was that HMME conjugated with Au@TiO_2_ showed better PDT effects than that of HMME alone on KB cells, which may due to the higher efficiency intracellular delivery and the higher drug delivery rates of the Au@TiO_2_–HMME conjugates. At the same time, due to the existence of the local field enhancement effect, the photodynamic effect of Au@TiO_2_–HMME under optimal conditions was improved compared with that of pure HMME in cells.

As shown in Figure 3, all the Au@TiO_2_ core-shell nanostructures had the LSPR absorption peak of the Au core, and the absorption spectrum of Au@TiO_2_–HMME conjugates proved the successful loading of HMME on the Au@TiO_2_. The Au@TiO_2_–HMME conjugates exhibited better inhibitory effect of KB cells under the LED at its SPR absorption than the xenon lamp when using different light sources with the same power density, which is obvious in Figure 6. A reasonable explanation is that the synthetic AuNP@TiO_2_–HMME shows the maximum absorption rate at around 540 nm, which matches the LED with the central wavelength of 523 nm. At the same time, compared with the xenon lamp with the same power, the LED has a narrower band, and the power is mainly concentrated near the central wavelength, resulting in a better cell-killing effect.

TEM results (Figure 2) exhibited that the prepared Au@TiO_2_ core-shell nanostructures and Au@TiO_2_–HMME conjugates had good dispersion, surface topography and stability, and that their size is close to optimal for cell endocytosis. Figure 7 compared the photodynamic effects of AuNP@TiO_2_–HMME with different shell thickness under the same condition. The results showed that the shell thickness has no appreciable effect on the overall PDT efficacy. Furthermore, the sublet different therapy efficiency for different shell thickness is mainly due to the SPR effect of AuNPs. When the TiO_2_ layer thickness is 4 nm, the absorption peak of AuNP@TiO_2_ is 540 nm, matching the HMME characteristic peak at 536 nm, which can give full play to the SPR effect of gold nanomaterials. In addition, local field enhancement is dependent on the distance from the surface of gold nanoparticles [48], while for a TiO_2_ layer thickness of 18 nm, the particle size of about 50 nm is closest to the ideal size of endocytosis, which induced more HMME uptake by cells [49].

At the same light dose with a different wavelength, the killing effect of AuNP@TiO_2_–HMME is better than that of AuNR@TiO_2_–HMME, as shown in Figure 8. At the same wavelength, the AuNP conjugates have a much better killing effect than HMME alone, while for AuNR conjugates, the cell viability was almost as some as HMME alone. We suspect that this is related to the efficiency of endocytosis and the specific surface area of different core-shell nanostructures, with AuNP@TiO_2_ nanostructures having higher specific surface area and endocytosis efficiency. This result may indicate that the shape of nanoparticles does affect their interactions with cells, and that spherical and shorter rod particles may be more easily endocytosed by cells than longer rod nanoparticles [49,50]. On the other hand, HMME has a weaker absorption at 635 nm than 530 nm, and the 635 nm was not the surface plasmon resonance peak of the AuNR conjugate, which limited the SPR effect of the conjugate. However, the results demonstrated that the AuNR has weaker drug delivery efficiency than AuNP in this study. On the whole, our experiments have proved some good phenomena in that these designed Au@TiO_2_ nanostructures can also enhance the PDT reaction of the photosensitizer adsorbed on their surface with the help of plasmonic gold core under the SPR_L_ wavelength.

Our study showed Au@TiO_2_–HMME conjugates could be a potential photo-agent for the treatment of skin cancer and oral mucosa cancer. However, in the subsequent research, the photodynamic experimental parameters of the conjugate Au@TiO_2_–HMME data on KB cells would be measured in greater detail to find the optimal photodynamic treatment condition by adjusting the shell thickness and HMME concentration. On the other hand, it is a good attempt to give full play to the high catalytic activity of anatase and improve the killing effect of the combination on cells by converting amorphous TiO_2_ into ideal anatase after calcination. This study completed the preparation and modification of gold nanoparticles, the preparation of conjugate which consisted of Au@TiO_2_ core-shell nanostructures with HMME and the cell experiments of the Au@TiO_2_–HMME conjugate. By means of the transmission electron microscope, ultraviolet visible light spectrophotometer and others, the size, morphology, absorbance and other characteristics of the synthetic material were characterized and analyzed, and the formation of stable conjugate was observed. Based on the above experiments, we could find that the conjugate had a greater effect on cell activity than HMME at the same concentration alone. For more remarkable performance of AuNP@TiO_2_–HMME, the high specific surface area of TiO_2_ could promote more HMME molecules to enter the cell, leading to an improvement in the photodynamic effect. Under the irradiation of 532 nm LED, the photodynamic effect of AuNP@TiO_2_–HMME with 4 nm or 18 nm core-shell thickness was better. The better realization of the photodynamic effect of HMME is affected by the wavelength of the irradiation light source and the plasma absorption peak of the Au@TiO_2_ core-shell nanostructure drug carriers.

## Data Availability

The data presented in this study are available on request from the corresponding author.
